# Monitoring and Assessment of Indoor Environmental Conditions in Educational Building Using Building Information Modelling Methodology

**DOI:** 10.3390/ijerph192113756

**Published:** 2022-10-22

**Authors:** Antonio J. Aguilar, María L. de la Hoz-Torres, Diego P. Ruiz, Mª Dolores Martínez-Aires

**Affiliations:** 1 Department of Applied Physics, University of Granada, Av. Severo Ochoa s/n, 18071 Granada, Spain; 2 Department of Building Construction, University of Granada, Av. Severo Ochoa s/n, 18071 Granada, Spain

**Keywords:** building information modelling, COVID-19, educational building, indoor environmental quality, sensor monitoring

## Abstract

Managing indoor environmental quality (IEQ) is a challenge in educational buildings in the wake of the COVID-19 pandemic. Adequate indoor air quality is essential to ensure that indoor spaces are safe for students and teachers. In fact, poor IEQ can affect academic performance and student comfort. This study proposes a framework for integrating occupants’ feedback into the building information modelling (BIM) methodology to assess indoor environmental conditions (thermal, acoustic and lighting) and the individual airborne virus transmission risk during teaching activities. The information contained in the parametric 3D BIM model and the algorithmic environment of Dynamo were used to develop the framework. The IEQ evaluation is based on sensor monitoring and a daily schedule, so the results show real problems of occupants’ dissatisfaction. The output of the framework shows in which range the indoor environmental variables were (optimal, acceptable and unacceptable) and the probability of infection during each lecture class (whether or not 1% is exceeded). A case study was proposed to illustrate its application and validate it. The outcomes provide key information to support the decision-making process for managing IEQ and controlling individual airborne virus transmission risks. Long-term application could provide data that support the management of ventilation strategies and protocol redesign.

## 1. Introduction

Nowadays, one of the goals of contemporary society is to achieve sustainable development and performance of the built environment [1]. Buildings have to not only meet the required standards for an indoor environment but also meet the occupants’ needs and ensure their satisfaction (including social, economic and environmental aspects) [2]. Since people are always surrounded by a physical environment, maintaining comfort, wellbeing and health poses a great challenge [3]. The role of indoor environmental conditions in buildings is essential, since people tend to spend 90% of their time indoors [4]. Such is its importance that previous research has even stated that indoor environmental quality (IEQ) is key in determining the success or failure of buildings [5]. Building occupants interact with their surrounding environments, and their feedback, as building users, helps determine the requirements for a comfortable IEQ [6]. Accordingly, if the indoor environment ensures up to 80% satisfaction of the occupants, the built environment can be assumed to be performing well [4,7].

However, buildings do not always meet the required indoor environmental conditions, which has a negative impact on their users [8]. These circumstances are critical in educational buildings, where students, teachers and staff spend long periods of the day. Primary and secondary school students spend more time at school than any other building except at home [9]; some studies claim that they spend around one-third of their day inside school [10]. In contrast, university students spend less time in classrooms (at least 3 h a day) compared to the two other educational stages [9]. Thermal, acoustic and lighting sensations are influenced by physical variables such as temperature, sound pressure level (SPL) and lighting, and therefore, overall satisfaction is also influenced. Previous studies have shown that poor IEQ in educational buildings is common and adversely influences the attendance, performance and health of students [11,12]. In addition, the recent events caused by the COVID-19 pandemic highlighted that a suitable IEQ is essential in educational buildings. Minimising the transmission of the SARS-CoV-2 virus led governments to implement measures to ensure that students could use spaces safely. For example, the measures introduced by the Spanish government included reducing contact time, distance social distancing and using facemasks in educational buildings. Furthermore, the ventilation rate (VR) inside the classroom had to be increased by continuous ventilation [13]. However, since most educational buildings in European countries are naturally ventilated, achieving high values of VRs (e.g., 6–10 ACH) is hard through a natural ventilation strategy [14]. In this context, several researchers have assessed if the VR required by governments for educational buildings could be achieved through natural ventilation strategies during the pre-pandemic scenario [7,15,16] and during the post-pandemic scenario [17,18,19,20,21]. Although the results presented by these studies showed that a continuous natural ventilation strategy could provide effective air renewal, they also showed that other indoor environmental variables were influenced. Wind speed and outdoor temperatures are critical variables that significantly affect the indoor environmental conditions if doors and windows are continuously opened. In addition, students who are seated close to windows are more exposed to drafts. Moreover, indoor acoustic quality can be affected by outdoor noise [22]. As a result, students’ comfort, health and productivity could contribute to a poor IEQ [11].

This situation poses a challenge to building facility managers who have to analyse and manage operation strategies to ensure that indoor spaces are safe for students and to guarantee a suitable IEQ. The Federation of European Heating, Ventilation and Air Conditioning Associations (REHVA) recommended installing CO_2_ sensors to evaluate the effectiveness of ventilation [14]. However, the calculation of the individual COVID-19 infection risk depends on more variables, including the ACH, type of activity conducted during the class, time of exposure, and size and occupancy of the room. Managing this information in a non-centralised database is complex and time-consuming.

Nevertheless, new methodologies are emerging in the architecture, engineering, construction and operation (AECO) sector as building information modelling (BIM). BIM is a methodology that offers new challenges and opportunities in the process of design, construction and maintenance of buildings [23,24]. BIM covers all phases of the building life cycle, and previous studies have shown its potential application during the building operation phase. Marzouk and Abdelaty [25] proposed the use of BIM to monitor IEQ in subway stations. Cheung et al. [26] developed a system to integrate a hazardous gas sensor network into BIM methodology. Nojedehi et al. [27] defined a methodology to integrate maintenance management systems and BIM to improve building management. Alavi et al. [28] proposed a probabilistic approach to evaluate occupants’ comfort using BIM. Artan et al. [29] defined a BIM-integrated post-occupancy evaluation system for office buildings. In this sense, this study starts from the premise that the integration of data obtained from indoor environmental monitoring into the BIM model could facilitate the process of data collection and subsequent analysis. However, to the best of our knowledge, no previous study has developed a framework for integrating occupants’ feedback in order to conduct the assessment of the individual airborne virus transmission risk and IEQ. This study aims to fill this research gap. In this context, the main objective of this study is to develop a framework based on BIM methodology for integrating the assessment of the IEQ and airborne virus transmission risk in higher educational buildings. To achieve this objective, a methodology has been defined to obtain the occupants’ feedback and integrate it into the BIM model. Subsequently, a BIM-based framework has been developed as a platform that can be used to support decision making by building managers and improve operational strategies. Finally, the proposed framework has been applied to a university building case study.

## 2. Material and Methods

### 2.1. Research Approach

**Figure 1:**
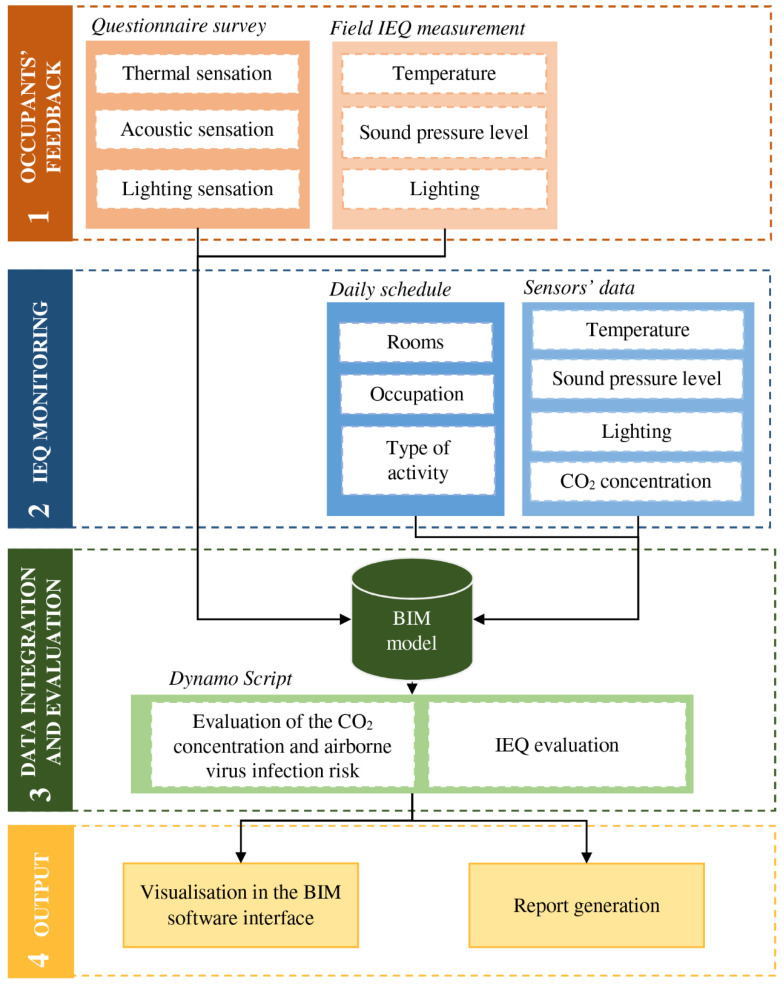
Automation process of integrating indoor environmental assessment and occupants’ feedback into BIM.

The proposed framework is based on four main phases: (1) obtaining occupants’ feedback on IEQ; (2) monitoring of the building’s IEQ variables; (3) integration of the data obtained in phases 1 and 2 into the BIM model and performing the evaluation; and (4) visualising the results in the BIM software interface and generating reports. Figure 1 shows the automation process of integrating the indoor environmental assessment and occupants’ feedback into BIM. The following subsections show the materials and methods used in each of the phases.

**Phase 1: occupants’ feedback.** The occupant comfort in existing buildings is directly determined from indoor environmental measurements and the responses of occupants through a questionnaire survey. The questionnaire used in this study was divided into two parts and was based on the recommendations for the evaluation of indoor environmental quality stated in UNE-CEN/TR 16798-2:2019 and UNE-EN ISO 28802:2012 standards [30,31]. The first part of the questionnaire was related to personal information (age, gender and clothing), while the second part contained questions related to physical environmental parameters. A 7-point Likert scale was used to evaluate the occupants’ indoor environmental perception: the thermal sensation vote (TSV, from −3 for ‘cold’ to 3 for ‘hot’), the acoustic sensation vote (ASV, from −3 for ‘very noisy’ to 3 for ‘very quiet’) and the lighting sensation vote (LSV, from −3 for ‘very bright’ to 3 for ‘very dark’). In addition, occupants were asked whether they were satisfied with the indoor thermal, acoustic and lighting environment, with a scale ranging from −3 (‘very dissatisfied’) to 3 (‘very satisfied’), with 0 being neutral.

Simultaneously with the survey, the measurement of indoor environmental variables was carried out. For this purpose, sensors were placed to record the radiant temperature, air temperature, air velocity, lighting, relative humidity (RH), CO_2_ concentration and SPL. Table A1 in Appendix A shows the sensors used to measure the indoor environmental conditions in the classrooms.

**Figure A1:**
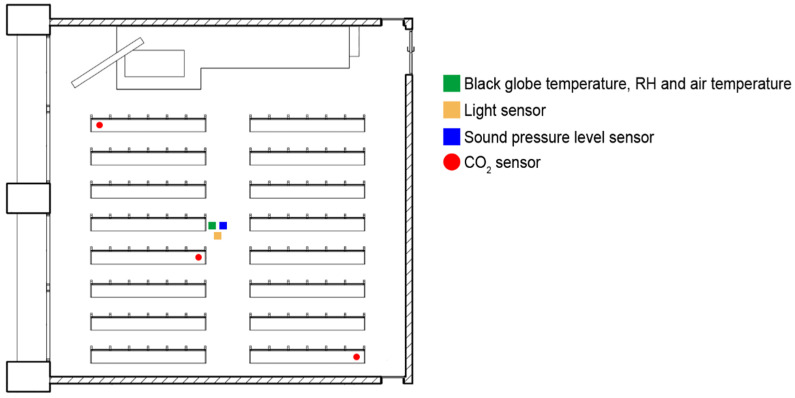
Layout of the sensor location in classroom.

**Phase 2: IEQ monitoring.** In this phase, the indoor environmental conditions in classrooms are monitored during teaching activities. The indoor environmental variables are monitored continuously at 1 min logging intervals. The sensors should be placed in the middle of the classroom at 0.6 m above floor level, following the recommendations in ISO 7726:1998. Figure A1 in Appendix A shows an example of the layout of the sensor location. The sensors indicated in Table A1 in Appendix A were used for this purpose. In addition, the occupation and the type of activity conducted in the classrooms are considered. These parameters are taken into account due to their impact on the occupants’ IEQ perception and the airborne virus infection risk.

**Phase 3: IEQ assessment and evaluation of airborne virus infection risk.** The data obtained in phases 1 and 2 and the geometric data contained in the BIM model are used as inputs in this phase. Subsequently, the evaluation process is divided into two parts.

Firstly, the evaluation of the airborne virus infection risk is carried out. In this regard, the measures implemented by governments to contain COVID-19 virus transmission in public buildings during the pandemic period are considered [13]. The probability of infection (P) due to the SARS-CoV-2 virus during teaching activities in educational buildings is calculated using the Wells–Riley airborne disease transmission model (see Equation (1)) [32].

(1) P=CiCs=1−e−I q p tACH

where $C_{i}$ is the number of occupants who develop an infection, $C_{s}$ is the number of susceptible occupants, I is the number of infectors, q is the quantum generation rate (h^−1^), p is the pulmonary VR of susceptible people, t is the exposure time (h), and ACH is the air changes per hour (h^−1^). This model assumes a steady-state infectious particle concentration that varies with the VR and a well-mixed room air. Therefore, the ventilation of indoor spaces is a key factor to control the airborne virus transmission.

One of the parameters used to monitor the VR and indoor air quality in the classroom is the CO_2_ concentration. The methods using CO_2_ as a tracer gas to estimate VRs are based on a fully mixed mass balance model (Equation (2)):

(2) $$V\frac{dC}{dt}=E+Q\cdot C_{outdoor}-Q\cdot C$$

where V is the volume of the indoor space under study, E is the CO_2_ emission rate of the occupants per hour, Q is the outdoor air flow rate, $C_{outdoor}$ is the CO_2_ concentration in the outdoor air, and C is the CO_2_ concentration in the indoor space. 

The approach proposed in this study relies on the assessment of the probability of airborne virus infection and the effectiveness of ventilation based on the ACH assessment for each scenario. For this purpose, the build-up VR technique is used. This technique uses the series of measurements of CO_2_ concentration over time with a solution to the fully mixed model (Equation (3)):

(3) $$C_{t}=\left(C_{S}-C_{outdoor}\right)\left(1-e^{-\mathrm{ACH}\;t}\right)+C_{outdoor}\;$$

Equation (4) may be linearised as the following expression (Equation (4)):

(4) $$\ln\left(C_{S}-C_{t}\right)=-\mathrm{ACH}\;t+\ln\left(C_{S}-C_{outdoor}\right)$$

Therefore, the ACH can be calculated as the slope of the $\ln\left(C_{S}-C_{t}\right)$ versus time t. The ACH derived using this method assumes that the VR is constant over a specific time period, and it is applied only to a single and fully mixed zone. For this purpose, the CO_2_ measurement sequence over time is required, from which the ACH is calculated by minimising the squared residuals. The build-up method has been used by previous studies to obtain the VR in an occupied classroom [33,34,35].

Secondly, the IEQ evaluation is conducted. For this purpose, the feedback obtained from the first phase is used to determine if the students are satisfied with the indoor environmental parameters. To this end, the indoor variables measured during the teaching activity in the classroom are compared with the values indicated as suitable by the occupants (in this case, the students).

**Phase 4: output generation.** The results obtained in phase 3 are shown to the facility managers in two different formats: (1) the results are shown in the same interface as the BIM software and (2) a report. The first one is shown automatically in the BIM software interface after the application of the framework. This format shows the results in two different charts (the first one shows the assessment of IEQ for each lecture, and the second one shows the probability of infection). In addition, the report is automatically generated in .csv format. It contains all the data obtained from the evaluation.

### 2.2. Case Study

**Figure A2:**
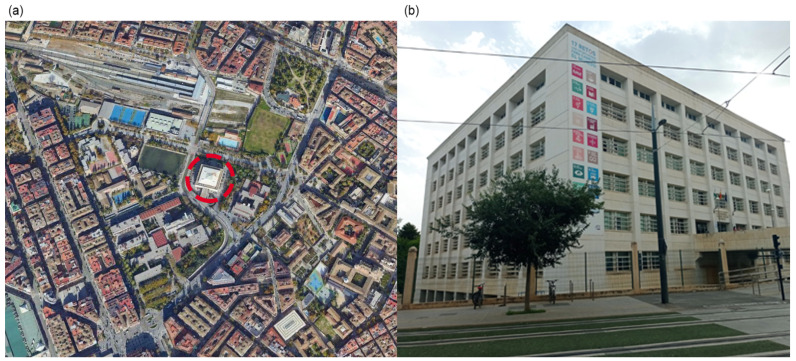
Polytechnic building of University of Granada: (**a**) location, (**b**) façade. Red dotted line indicates the location of the polytechnic building.

The polytechnic building of the University of Granada was used as a case study to evaluate the proposed BIM framework (Figure A2 in Appendix A). The building is located at the Fuentenueva campus, in the urban area of the city of Granada. Granada is characterised by a climate classified as Csa according to the Köppen–Geiger climate classification. The temperature is generally in the range 0–34 °C and rarely rises above 38 °C or drops below −4 °C during the course of the year.

The polytechnic building was built in 2000 and has seven floors with a concrete structure (waffle slab), concrete wall and flat roof with a sloping section. Classrooms are naturally ventilated and have heating systems (radiators). Table 1 shows the main characteristics of the classrooms.

The occupants’ feedback survey was conducted during the academic year 2021–2022 (four times a month, 10 months from September 2021 to June 2022). The questionnaires were filled out by students in middle morning or middle afternoon, during the last fifteen minutes of the lecture class. This decision was intended to minimise the lecture disturbance and to maximize the exposure of the students to the indoor environmental conditions (students had been sitting for at least 1 h in the classroom). This field study followed the recommendations stated in UNE-CEN/TR16798-2:2019 and ISO 10551:2019 [30,36]. A total of 930 responses were obtained.

### 2.3. Statistical Analysis

A statistical analysis was carried out with the data obtained from the field measurement campaign. The subjective data collected in combination with the objective data obtained from the sensors’ field measurements were used to relate the occupants’ perception to the indoor environmental conditions of the buildings. To evaluate the IEQ affecting the building occupants, regression methods were applied to the related objective and subjective results obtained. SPSS software (v.23.0) was used to perform this analysis.

## 3. Integration of the Proposed Methodology into BIM

**Figure 2:**
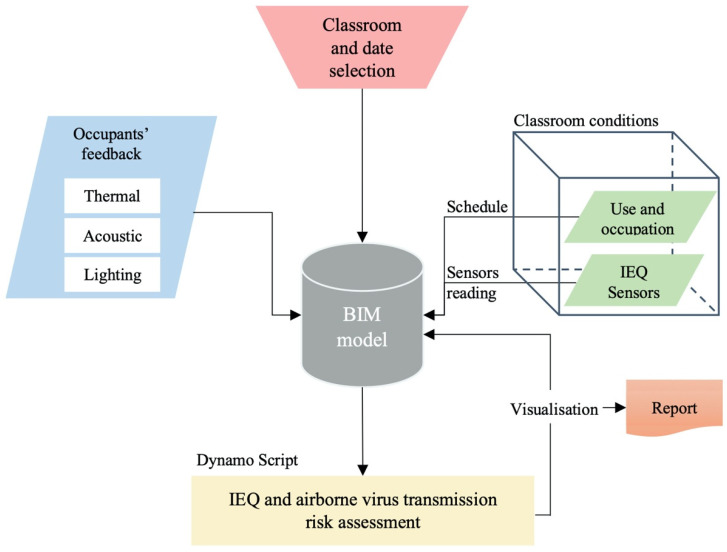
Workflow of the system developed in BIM.

As indicated in the previous section, a framework was developed to integrate the occupants’ feedback and the data obtained from the sensor monitoring into the BIM model. In this study, the software BIM Revit^®^ (v. 2023) and its open-source visual programming language Dynamo (v. 2.13) were used. Figure 2 shows the workflow of the system developed in the BIM. Firstly, a BIM model of the building under study was required to implement the developed framework. This model had to incorporate all the geometric and non-geometric information of the building. Therefore, a BIM level of detail (LOD) 300, as the minimum, was required to run the assessment process.

In addition, several shared parameters needed to be defined during the design of the BIM model (Table 2). These parameters were used to store and communicate information about the components of the BIM model.

**Figure 3:**
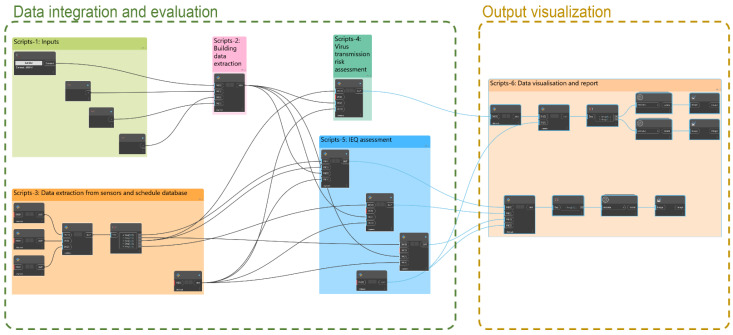
Overview of the diagram of the scripts developed using Dynamo.

The proposed system developed in BIM included six main Dynamo packages or scripts, which are classified as follows (Figure 3):

- Scripts-1: Inputs.
- Scripts-2: Building data extraction.
- Scripts-3: Data extraction from sensors and schedule database.
- Scripts-4: Virus transmission risk assessment.
- Scripts-5: IEQ assessment.
- Scripts-6: Data visualisation and report.

The set of Scripts-1 Inputs allowed the selection of the classroom under study as well as the date considered for the evaluation. This information was used to identify the indoor environmental conditions of the selected classroom during the period under study. The classroom was selected directly in the BIM model. Subsequently, the period of analysis (start and end date) was entered through a string.

Scripts-2 Building data extraction obtained the geometric data (dimensions, volume and area) and the non-geometric data (Classroom_Id, Max_occup and ID_sensors) of the selected classroom.

The next set, Scripts-3 Data extraction from sensors and schedule database, connected and imported the data obtained from the sensors placed in the classroom (temperature, lighting, SPL and CO_2_ concentration) for the period under study. The parameter ID_sensors was used to link the sensor located in each classroom with the virtual model of the building. In addition, this process also imported the schedule of teaching activities that were conducted in the classroom during the selected period. The information imported for each teaching activity is shown in Table 3.

Subsequently, based on the information incorporated by the previous sets of scripts, the assessment of the virus transmission risk was carried out in Scripts-4. For this purpose, the methodology defined in Section 2 was applied, and the probability of infection due to the SARS-CoV-2 virus was calculated using the Wells–Riley airborne disease transmission model (Equation (1)). According to the type of activity carried out during the teaching activity, the breathing rate of the students and professors (p) was selected: lectures where students were seated (p = 0.50 m^3^/h), laboratory activities where students were carrying out experiments (p = 0.65 m^3^/h), etc. These breathing rate values were obtained from REHVA COVID-19 guidance [14]. 

In addition, since the probability of transmission of the SARS-CoV-2 virus (Omicron variant) was considered in this study, two possible scenarios were evaluated when selecting the quanta emission: (1) q = 4.0 quanta/h, assuming that there is an infected student ($C_{i}$ = 1), and (2) q = 10.8 quanta/h, assuming that the infected person is the professor ($C_{i}$ = 1) [37]. However, if another variant or airborne virus transmission is assessed, the model will have to be modified and the appropriate quanta emission rate selected. The number of susceptible occupants ($C_{s}$) was equal to the classroom’s occupation minus the number of infected occupants. The pulmonary VR was assumed as 0.54 m^3^/h for students and 0.65 m^3^/h for the professor [37]. The exposure time (t) was equal to the duration of each lecture. This script automatically evaluated, as a function of the occupation pattern obtained from the schedule database and the CO_2_ time series measured by the sensors, the VRs during each class (ACH). Equation (4) (shown in Section 2) was used for this purpose. The ACH obtained value was used to calculate the probability of airborne virus transmission during each class.

Parallel to this process, the set of Scripts-5 assessed the indoor environmental conditions. The occupants’ feedback obtained from the field survey (TSV, ASV and LSV) was analysed alongside the indoor environmental variables measured simultaneously (temperature, SPL and lighting), and the comfort zones were identified for each variable. In the case of thermal comfort, the ANSI/ASHRAE 55-2020 and the ISO 7730 standards [4,38] were followed according to the comfort zone method. These standards state that, to maintain a percentage of dissatisfied occupants below 10%, the TSV should be between +0.5 and −0.5, while to maintain it below 20%, the TSV should be between +1 and −1. Therefore, three thermal comfort ranges were established (Equation (5)) [4]:

(5) $$\begin{array}{ccc} a. & -0.5<TSV<0.5 & Optimum\\ b. & -1.0<TSV<1.0 & \;\;Acceptable\\ c. & TSV\langle -1.0\;\;or\;\;TSV\rangle 1.0 & \;\;\;\;\;\;\;Unacceptable \end{array}$$

The same strategy was assumed for the indoor light quality (Equation (6)):

(6) $$\begin{array}{ccc} a. & -0.5<LSV<0.5 & Optimum\\ b. & -1.0<LSV<1.0 & \;\;Acceptable\\ c. & LSV\langle -1.0\;\;or\;\;LSV\rangle 1.0 & \;\;\;\;\;\;\;Unacceptable \end{array}$$

However, regarding the indoor acoustic quality, this study evaluated the indoor background noise to conduct the assessment. In this case, since the objective was to keep the background noise SPL at a level that did not interfere with the teaching learning activity, the three ranges were assumed as follows (Equation (7)):

(7) $$\begin{array}{ccc} a. & 0.5<ASV & Optimum\\ b. & 0<ASV<0.5 & \;\;Acceptable\\ c. & \;ASV<0 & \;\;\;\;\;\;\;Unacceptable \end{array}$$

The final step of the system generated in Dynamo was Scripts-6 Data visualization and report. This set of nodes made it possible to visualise the data obtained from the assessment process in the same BIM software, as well as to export a report of these data in a .csv format file. With respect to the IEQ assessment, the data are displayed in a stacked bar chart with the results obtained. The diagram shows the comfort zone and, for each time slot of the classroom under study, the thermal, lighting and acoustic conditions. The python library matplotlib is used for this purpose. In addition, with respect to the results obtained from the virus transmission risk assessment, a percentage of probability of infection is plotted using a range of colours depending on the value obtained.

## 4. Results: Case Study

### 4.1. Occupants’ Feedback Survey

**Figure 4:**
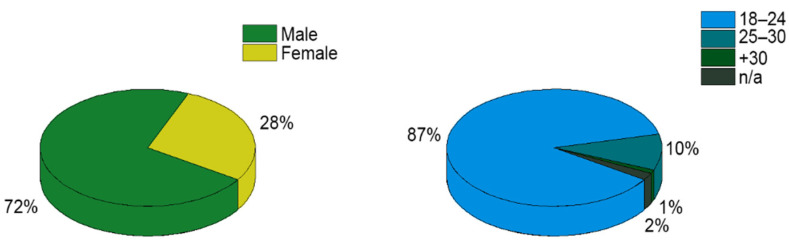
Gender and age of the surveyed students.

The occupants’ feedback survey and IEQ measurement were carried out simultaneously during the 2021/2022 academic year. A total of 930 questionnaires were collected in this study, of which 908 were valid (22 were incomplete). Therefore, the feedback from 908 students was analysed and subsequently incorporate into the BIM model. The participants (university students) were sitting and listening to the lecturers during the field measurements. Figure 4 shows the distribution of the respondents’ general information.

Regarding the participants’ characteristics, 87% were aged between 18 and 24, a figure that rose to 97% when participants aged between 25 and 30 were included. The results obtained from the sensor monitoring are summarised in Table 4.

**Figure 5:**
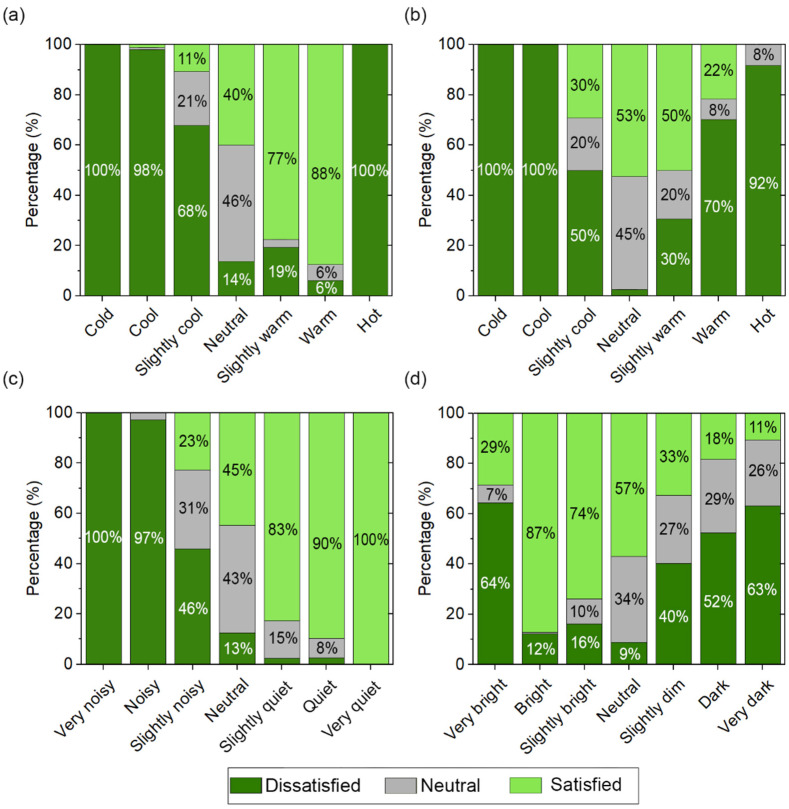
Sensation votes and satisfaction votes: (**a**) thermal (heating season), (**b**) thermal (non-heating season), (**c**) acoustic, (**d**) lighting.

Figure 5 shows the relationships between the sensation vote values and satisfaction vote values. In terms of indoor thermal quality during heating season (Figure 5a), the values obtained show that between 98% and 100% of the students who voted that the indoor thermal conditions were cold/cool or hot were dissatisfied. The values obtained of indoor thermal quality during the non-heating season (Figure 5b) show that between 92% and 100% of the students who voted that the indoor thermal conditions were cold/cool or hot were also dissatisfied, while dissatisfaction dropped to 70% for warm TSVs. These values may indicate a greater adaptation to warm environments than to cold environments. Figure 5c shows the relationship of student acoustic satisfaction and ASV. These values reveal that for a ‘very noisy’/’noisy’ ASV, 100–97% of students were dissatisfied, while for a ‘slightly noisy’ ASV, the percentage of dissatisfied students dropped to 46%. With respect to the indoor lighting quality (Figure 5d), about 63–64% of students who indicated dissatisfaction also indicated a ‘very bright’ or ‘very dark’ LSV. In fact, it was the less well-lit environments that caused the most dissatisfaction among students.

**Figure 6:**
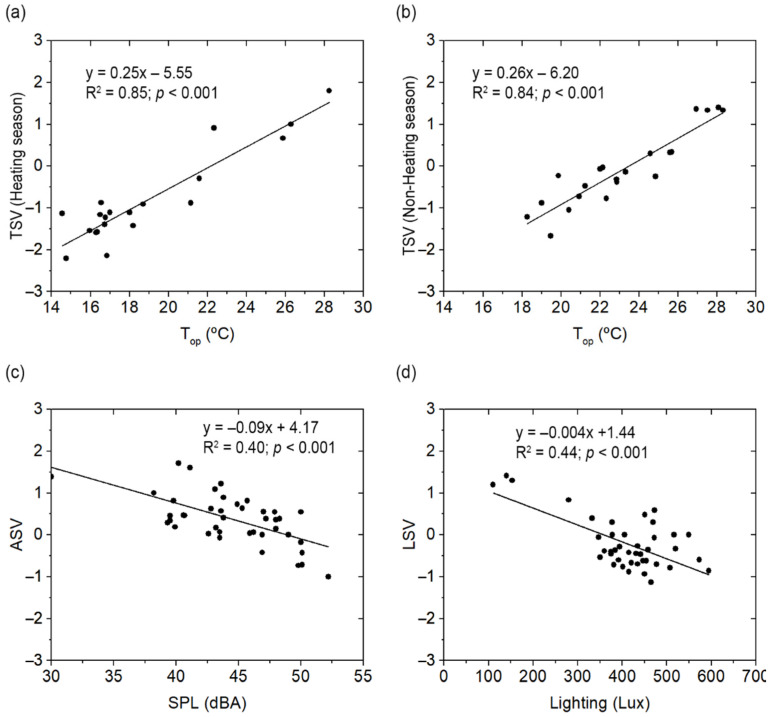
(**a**) TSV vs. T_op_ winter season; (**b**) TSV vs. T_op_ summer season, (**c**) ASV vs. SPL, (**d**) LSV vs. lighting.

Moreover, with respect to the relationship between the objective variables and sensation votes for each of the environmental factors studied, the results obtained are shown in Figure 6. Since the thermal adaptation and clothing level affect TSV, the relationship between the temperature and the subjective thermal perception of students was analysed separately for the winter and summer seasons. Figure 6a,b show the results obtained for the heating season (R^2^ = 0.85, *p* < 0.001) and non-heating season (R^2^ = 0.84, *p* < 0.001), respectively. It was found that the neutral temperature was 22.2 °C for the winter season and 23.5 °C for the summer season. Figure 6c shows the relationship between the LSV and the lighting values (R^2^ = 0.44, *p* < 0.001). In addition, Figure 6d shows the relationship between the ASV and the background noise measured in the classrooms (R^2^ = 0.40, *p* < 0.001). Based on the equation obtained from this regression analysis, the comfort zone was defined (Table 5). The sensation votes equal to ‘−1’, ‘−0.5’, ‘0’, ‘0.5’ and ‘1’ were calculated for each environmental factor.

### 4.2. Implementation in the BIM Model

**Figure 7:**
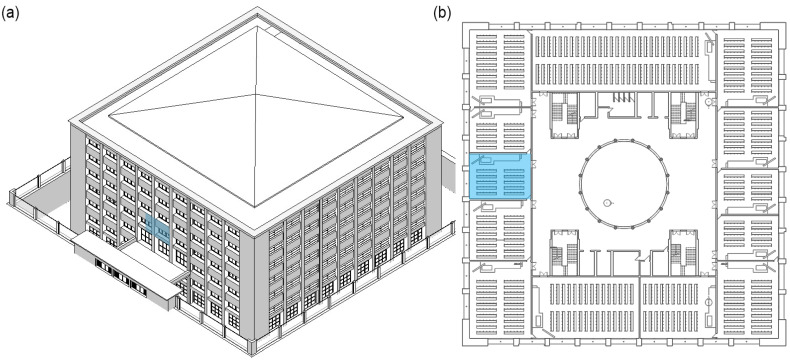
(**a**) BIM model of polytechnic building. (**b**) Plan of the first floor. Blue colour indicates classroom 110.

This section shows an example of the application of the system developed in BIM to one of the classrooms of the building under study. Classroom 110 was selected on 30 May 2022 (non-heating season), whose teaching activity time schedule is shown in Table 6. Figure 7 shows the classroom selected.

**Figure 8:**
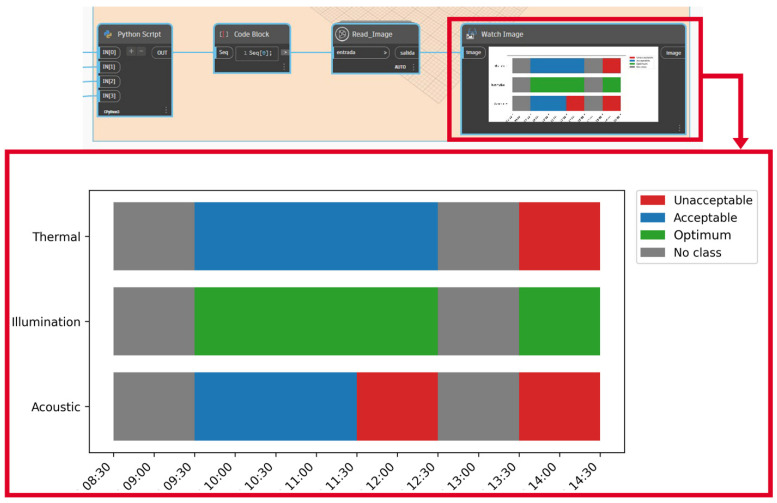
IEQ evaluation results of classroom 110.

Protocols in this educational building include that classes must end ten minutes before the scheduled time to provide a rest interval for students and renew the air in the classroom. The results obtained from the IEQ evaluation in classroom 110 are shown in Figure 8 and Table 7. Green, blue and red colours indicate that the variables are in the optimum, acceptable and unacceptable ranges, respectively.

In the analysed case, the indoor thermal conditions were acceptable during the first two lectures (the mean indoor temperature was 24.9 °C and 25.2 °C during Sanitary Engineering Group A and B lectures, respectively) and unacceptable during Structural analysis (27.8 °C, higher than the 26.2 °C limit value of the acceptable comfort zone). It should be noted that the last lecture took place during the warmest hours of the day, which, together with the fact that it was the most occupied lecture class, resulted in a higher indoor temperature.

Regarding indoor acoustic conditions, these were unacceptable during Sanitary Engineering (Group B) and structural analysis, and acceptable during Sanitary Engineering (Group A). Since this building is located in the urban area of Granada, natural ventilation strategies affect the indoor environmental conditions: outside urban noise (e.g., disturbing traffic noises such as sirens, trucks, etc.) and people talking in common areas (e.g., corridors) affect the acoustic conditions. In contrast, the indoor lighting conditions were optimal during all the lectures (all the mean values were in the optimum range). 

**Figure 9:**
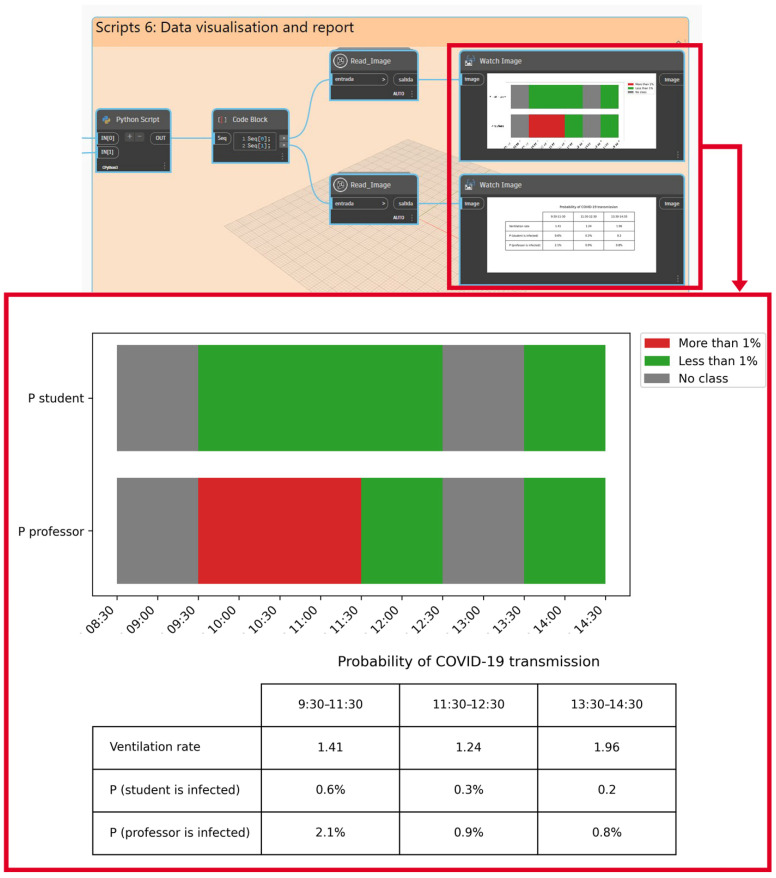
Assessment of the probability of airborne virus transmission in classroom 110.

In addition, the VRs inside classroom 110 during the teaching activities were calculated, and the probability of COVID-19 virus transmission was estimated assuming two scenarios: (1) a student was infected, and (2) the professor was infected. In addition, Figure 9 is plotted in the Dynamo environment. The results obtained for this case indicated that the probability exceeded 1% only during the first lecture (9:30–11:30) and assuming the scenario where the infected person was a professor. This lecture had the longest exposure time (2 h) and the second lowest ventilation rate. These conditions, together with the fact that the professor’s quantum generation rate was higher (they talked more during the lecture), determine that the probability of COVID-19 infection is higher in this scenario (above 1%) than during the rest of the lectures.

## 5. Discussion

### 5.1. Field Measurement Campaign

In relation to the occupants’ feedback survey, since the statistics published by the University of Granada in the report for the academic year 2020/2021 indicated that 95% of students on university degrees were aged between 18 and 30 [39], the values shown in Figure 4 were to be expected. With regard to the operative temperature during the heating season, the measured values ranged from 14.5 °C to 26.3 °C, and the RH ranged from 26.9% to 49.4%. In contrast, during the non-heating season, the operative temperature ranged from 19.1 °C to 28.3 °C, and the RH ranged from 21.3% to 50.1%. Regarding air velocity, this variable ranged between 0.01 and 0.15 m/s and 0.01 and 0.22 m/s during the heating and non-heating seasons, respectively. These environmental factors were influenced by the continuous natural ventilation through the opening of doors and windows [40]. Both operative temperature and RH reached values that were out of the ranges defined by the Spanish state regulation [41] (21–23 °C and 40–50% in heating season, and 23–25 °C and 45–60% in the non-heating season). These obtained results from the field measurement campaign were similar to those found in other studies conducted during the pandemic scenario. In fact, other studies conducted in Spain found that there was a significant period with out-of-range temperature conditions in natural ventilated classrooms during the COVID-19 pandemic [19,42]. Regarding CO_2_ concentration, values between 400 ppm and 1676 ppm were recorded. Some of these concentration values were above the limits indicated by the Spanish regulations (900 ppm), as well as exceeding the REVHA recommendations for educational buildings for indoor climate during the COVID-19 pandemic (800 ppm). Similar CO_2_ concentration values were reported by studies conducted in classrooms where natural ventilation strategies were implemented [42,43]. With regard to the indoor acoustic quality, values between 30.0 dBA and 52.2 dBA SPL were observed. Since background noise is an essential factor in educational buildings due to its effects on the quality of the learning process, previous research considered that the level of 35 dBA should not be exceeded in order to guarantee good speech intelligibility [44]. In this sense, the natural ventilation protocols implemented because of the COVID-19 pandemic have also influenced the indoor acoustic quality inside classrooms, increasing the background SPL [22]. The opening of doors and windows can increase the background noise level in the classroom, as it decreases sound insulation with neighbouring spaces (traffic noise, people talking in neighbouring areas, etc.). In the case of lighting, the measured values ranged from 110 to 594 lux. The UNE-EN 16798-1:2020 standard [45] recommends an illumination of 500 lux for classrooms. Therefore, the low lighting values observed may affect students’ academic performance.

### 5.2. BIM-Based Framework

The obtained results from the case study evidence that the proposed framework can be used to assess the IEQ comfort requirements and the individual airborne transmission risk of COVID-19 in the BIM methodology. One of the advantages of the proposed framework that was identified is that the use of the BIM model of existing buildings together with its linkage to IEQ data allows the generation of a centralised database of the building characteristics. Therefore, the barrier to collecting the different information required is eliminated. The proposed methodology encourages the use of BIM models, which are currently mainly used in the design phase and throughout the whole of the building operation phase. In fact, the feedback obtained from groups of building occupants can be used by facility managers to evaluate indoor environmental conditions. The proposed methodology provides key information for post-occupancy IEQ evaluation and strategies for the control of ventilation. The framework automatically estimates the acoustic, lighting and thermal comfort of occupants based on the reading of sensor data. The results are shown in the software BIM interface, reducing the data processing time. The fact that all the information is available in a single database provides building managers with the necessary tool to analyse the facilities for any given period of time. This process provides valuable information for the building management and planning of the different subjects and classrooms. As a result, facility managers can use these results to support decision-making processes and improve building performance.

Moreover, the evaluation is based on occupant feedback, and the results therefore show real problems of occupants’ dissatisfaction. Facility managers can make decisions based on this information from an occupant-centric point of view. In fact, the proposed framework can be applied to other type of spaces (e.g., offices, library, labs, etc.) as long as the subjective responses of the occupants of the spaces under evaluation are obtained. Therefore, the building manager can choose the room to be analysed with the proposed framework and use the generated output to support the building management decision-making process. Furthermore, the methodology presented in this study not only provides information to assess whether indoor spaces are safe for occupants (by assessing the risk of airborne virus infection), but also assesses whether the protocols implemented result in poor indoor environmental conditions. Therefore, the obtained results also show the potential use of the proposed framework and BIM methodology. This fact is in line with the conclusions provided by previous studies applying BIM and field environmental measurement data during the operational phase of the building. For example, Chang et al. [46] developed a BIM-based platform using Dynamo to visualize sensor data in BIM and help in making energy-saving management decision. Valinejadshoubi et al. [47] evaluated the applicability of BIM for an efficient sensor failure management system during the operational phase of a building and concluded that the information within BIM allows better and more effective decision making for building facility managers. In addition, Desogus et al. [48] tested the integrated use of BIM methodology and IoT systems using Dynamo, and concluded that the BIM model allows the management of useful information about the building, which is key for effective and accurate building management.

It should be highlighted that managing IEQ data has become critical in the aftermath of the COVID-19 pandemic, and ensuring the good ventilation of educational spaces is a challenge today. In this sense, this framework provides an analysis of data obtained from CO_2_ sensors and estimates the VR of each classroom. These data are used to assess the probability of infection by different airborne viruses, such as the SARS-CoV-2 virus. Regarding the probability infection results obtained from the case study, it should be noted that although Sanitary Engineering (Group A, 9:30–11:30) was the lecture that had the second lowest ventilation rate, it also had the longest exposure time (2 h), and the professor’s quantum generation rate was higher than that of the students (as professors spend more time talking during lectures). In fact, Equation (1) of the Wells–Riley model shows this relationship: infection rate increases as the exposure time increases, while infection rate decreases as ventilation rate increases. In contrast, the results obtained from the last lecture show the highest ventilation rate and a probability of infection of less than 1%. However, the outdoor environmental conditions and surrounding spaces have a greater influence on the indoor environmental conditions of the classroom (acoustic and thermal conditions are outside the range of acceptable zone values for both variables). In addition, the threshold for the probability of infection was fixed at 1% because previous studies that analysed natural ventilation strategies and COVID-19 transmission considered 1% as a reference value of infection probability [49]. Nevertheless, this percentage can be modified in the framework at the discretion of the building manager.

Finally, the long-term application of the proposed methodology could provide data that can support the evaluation of the ventilation and management strategies implemented in buildings. This information is crucial for redesigning protocols and minimising the impact of poor IEQ conditions on building occupants.

## 6. Conclusions

This research has developed a framework to assess IEQ and the risk of infection by airborne viruses integrated into the BIM environment. The proposed system has been applied to an educational building of the University of Granada for validation. The main contributions of this study are as follows:

- The framework allows the integration of IEQ parameters and models to evaluate thermal, light and acoustic comfort into the BIM model.
- The system automatically calculates the thermal, acoustic and light sensation values for each of the classes in the selected period. It also avoids the possibility of information losses and errors in the process of assessment. Furthermore, the results are visualised in the same interface of the BIM software, facilitating the identification and detection of possible problems in the classrooms.
- The proposed system is an effective tool for building managers to manage IEQ and control airborne virus transmission. Its implementation supports decision making by providing useful information for the continuous assessment of the building. In addition, it is worth noting that it allows the building to be evaluated continuously over time and can be applied at any time as long as the time series measured by the sensors are available, as well as the evaluation and preventive diagnosis of buildings.
- The results presented in the case study showed that the proposed framework is a useful tool for building managers. The framework allows the identification of indoor environmental conditions out of the comfort range, such as thermal conditions (during “Structural Analysis”) and acoustic conditions (during “Sanitary Engineering Group B” and “Structural Analysis”). It can also identify and visualise the risk of infection as shown in the scenario of an infected professor (with a probability of 2.1%).

## Figures and Tables

**Table 1:** Characteristics of the classrooms in the polytechnic building.

Finishing Materials	Type of Windows	Mean Area (m^2^)	Mean Occupancy Ratio (m^2^/Person)
Wall: Gypsum plaster/Ceramic tile	Aluminium glazed windows (sliding)	170 ± 40.5	1.6 ± 0.9
Floor: Natural stone			
Ceiling: Registrable suspended ceiling			

**Table 2:** Shared parameters used in the model.

Parameter	Definition	Parameter Type	Category
Classroom_Id	Name of the classroom	String	Room
Max_occup	Maximum number of occupants	Number	Room
ID_Sensors	Identifier number of each sensor located in the classroom	String	Room

**Table 3:** Characteristics of teaching activities.

Element	Definition	Data-Type
Classroom_Id	Name of the classroom where the teaching activity is carried out	String
Subject	Name of the subject	String
Start time	Day and time when the class starts	Date
End time	Day and time when the class ends	Date
Type	Type of teaching learning activity (e.g., lecture, laboratory class, etc.)	String
Occupation	Number of students attending the class	Integer

**Table 4:** Results obtained from the field measurements during heating season (HS) and non-heating season (NHS).

Type	Operative Temperature (T_op_) (°C)		RH (%)		Air Velocity (m/s)		CO_2_ Concentration (ppm)	Lighting (lux)	SPL (dBA)
	HS	NHS	HS	NHS	HS	NHS			
Max	26.3	28.3	49.4	50.1	0.15	0.22	1676	594	52.2
Min	14.5	19.1	26.9	21.3	0.01	0.01	400	110	30.0
Mean	18.4	23.6	38.3	37.7	0.04	0.04	592	409	44.6
Median	16.9	22.9	39.4	38.2	0.02	0.02	511	420	44.1
SD	3.3	3.0	6.5	7.8	0.05	0.05	233	101	4.3

**Table 5:** Values calculated for the comfort zones based on the sensation votes.

Parameter	Sensation Votes				
Values	−1	−0.5	0	+0.5	+1
Thermal (winter)	18.2 °C	20.2 °C	22.2 °C	24.2 °C	26.2 °C
Thermal (summer)	19.7 °C	21.6 °C	23.5 °C	25.4 °C	27.3 °C
Lighting	611 lux	486 lux	361 lux	236 lux	111 lux
Acoustic	-	-	48.9	43.0 dBA	37.2 dBA

**Table 6:** Schedule of the teaching activities in classroom 110.

Date	Time	Subject	Type of Activity	Occupation
30 May 2022	09:30–11:30	Sanitary Engineering (Group A)	Lecture	28
30 May 2022	11:30–12:30	Sanitary Engineering (Group B)	Lecture	30
30 May 2022	13:30–14:30	Structural analysis	Lecture	40

**Table 7:** Mean values of indoor environmental conditions.

Time	8:30–9:30	09:30–11:30	11:30–12:30	12:30–13:30	13:30–14:30
Temperature (°C)	24.1	24.9	25.2	25.6	27.8
Lighting (lux)	249	368	447	434	408
SPL (dBA)	40.3	44.5	50.1	44.3	49.5

## Data Availability

Data are provided upon request to the corresponding author.

## Appendix A

**Table A1:** Characteristics of the sensors used to measure the indoor environmental parameters.

Variable	Sensor	Range	Accuracy
Air temperature	FHAD 46-C41A AHLBORN	−20 to +80 °C	Typical ±0.2 K at 5 to 60 °C Maximum ±0.4 K at 5 to 60 °C Maximum ±0.7 K at −20 to +80 °C
Mean radiant temperature	FPA805GTS AHLBORN	–50 to 200 °C	0.1℃
Air velocity	HD403TS2 Delta OHM^®^	0.1 to 5 m/s	±0.2 m/s + 3% f.s
RH	FHAD 46-C41A AHLBORN	0 to 98% RH	±2.0% RH in range from 10 to 90% RH
CO_2_ concentration	HOBO^®^ MX1102	0 to 5000 ppm	±50 ppm ±5% of reading
Lighting	HOBO^®^ MX1104	0 to 167,731 lux	±10% typical for direct sunlight
SPL	Imperum-R TECNITAX^®^ Ingeniería	35 to 115 dBA	±1 dBA
